# EuroQol (EQ-5D) measure of quality of life predicts mortality, emergency department utilization, and hospital discharge rates in HIV-infected adults under care

**DOI:** 10.1186/1477-7525-5-5

**Published:** 2007-01-25

**Authors:** William C Mathews, Susanne May

**Affiliations:** 1 Department of Medicine, University of California San Diego, San Diego, CA, USA; 2 Division of Biostatistics and Bioinformatics, Department of Family and Preventive Medicine and Department of Neurosciences, University of California San Diego, La Jolla, CA, USA

## Abstract

**Background:**

Health-related quality of life (HR-QOL) is a relevant and quantifiable outcome of care. We implemented HR-QOL assessment at all primary care visits at UCSD Owen Clinic using EQ-5D. The study aim was to estimate the prognostic value of EQ-5D for survival, hospitalization, and emergency department (ED) utilization after controlling for CD4 and HIV plasma viral load (pVL).

**Methods:**

We conducted a retrospective analysis of HIV clinic based cohort (1996–2000). The EQ-5D includes single item measures of: mobility, self-care, usual activities, pain/discomfort, and anxiety/depression. Each item is coded using 3-levels (1 = no problems; 2 = some problems; 3 = severe problems). The instrument includes a global rating of current health using a visual analog scale (VAS) ranging from 0 (worst imaginable) to 100 (best imaginable). An additional single item measure of health change (better, much the same, worse) was included. A predicted VAS (pVAS) was estimated by regressing the 5 EQ-5D health states on VAS using reference cell coding of health states and random effects linear models. Survival models were fit using Cox modelling. Hospitalization and ED rate models were estimated using population-averaged Poisson models.

**Results:**

965 patients met eligibility criteria. 12% were female; 42% were non-white. Median time-at-risk was 1.2 years. Median CD4 was 233. Median log_10_(pVL) was 4.6. 47 deaths occurred. In two Cox models controlling for CD4 and pVL, the adjusted hazard ratios (aHR) for VAS and pVAS as time-varying covariates were 0.73 (95% CI: 0.63–0.83) and 0.66 (95% CI: 0.56–0.77) respectively, for every 10 point increase in (p)VAS rating. In Poisson regression models predicting ED visit rates and hospital discharge rates controlling for current CD4 and pVL, each of the EQ-5D health dimensions, VAS, and health change items were significantly (p < 0.05) associated with the outcomes. For ED visit rates, the adjusted incidence rate ratios (aIRR) were 0.86 (0.83–0.89) and 0.79 (0.75–0.82) for VAS and pVAS, respectively. For hospital discharge rates, the aIRR's were 0.85 (0.82–0.88) and 0.79 (0.75–0.82) for VAS and pVAS, respectively.

**Conclusion:**

EQ-5D is a brief and prognostically useful predictor of mortality, hospitalization, and ED utilization among adults under care for HIV infection, even after adjusting for CD4 and HIV plasma viral load.

## Background

Self-reported measures of health related quality of life (HR-QOL) and functional status have been widely incorporated in clinical trials and observational cohort studies but are infrequently included among measures routinely administered as part of primary care for patients with HIV infection. In 1996, we implemented routine assessment of HR-QOL using the EuroQol (EQ-5D) instrument at all urgent and routine visits to the UCSD Owen Clinic, a multidisciplinary adult HIV clinic. The EQ-5D is a brief, standardized, generic measure of HR-QOL that provides a profile of patient function and a global health state rating[1]. Previous work from the HIV Cost and Services Utilization Study (HCSUS) showed that a different measure of physical HR-QOL was prognostic for survival after adjustment for stage of HIV disease and CD4+ lymphocyte count[2]. The 27-item HCSUS HR-QOL instrument measured seven domains (physical functioning, role functioning, bodily pain, general health perceptions, emotional well-being, social functioning, and energy). We chose the EQ-5D because of its brevity, acceptability for routine and repetitive administration, and suitability for cost-utility studies both in HIV-infected patients and in patient populations with other disease conditions. We examined the longitudinal pattern of EQ-5D responses in our dynamic HIV health care cohort to assess its prognostic value for survival and measures of health care utilization. The primary study aim was to estimate the prognostic value of EQ-5D for survival, hospitalization, and emergency department (ED) utilization after controlling for CD4+ lymphocyte count and HIV plasma viral load. Secondary aims were: (1) to estimate the percent, and stability with repeated administration, of variance in the EQ-5D global health ratings (VAS) explained by responses to the five EQ-5D health dimensions; and (2) to validate previous observations that EQ-5D VAS can discriminate HIV-infected patients by disease severity.

## Methods

We conducted a retrospective analysis of a cohort of adults under care for HIV infection at the UCSD Owen Clinic between 1996–2000. Patients were eligible for inclusion if they had confirmed HIV infection and one or more primary care visits at the study clinic during the study period. The principal study measures were longitudinal responses to the EQ-5D and to an additional single item measure of health change. The instrument was administered by medical assistants at the time of clinic check-in following the recording of vital signs. Study covariates were documented in a longitudinal electronic medical record and included socio-demographic characteristics, CD4+ lymphocyte counts, HIV plasma viral load (Roche Amplicor HIV-1 Monitor), hospitalizations, emergency department visits, and mortality verified by search of the Social Security death index. EQ-5D includes single item measures of five health dimensions: mobility, self-care, usual activities, pain/discomfort, and anxiety/depression. Each item has three possible response options that allow the patient to ordinally (no problems/some or moderate problems/extreme problems) rate their current state with respect to each of the 5 domains. In addition, EQ-5D includes a global rating of current health using a visual analog scale (VAS) ranging from 0 (worst imaginable) to 100 (best imaginable). We also included a separate single item measure of health change (better, much the same, worse) compared to the prior year.

For each eligible patient, analysis time began with the first visit to the Owen Clinic or on 1 January 1996 for those with visits prior to that date. Analysis time was divided into six month intervals by calendar year. For each six month period in which a patient had more than one visit, median values of VAS and the five EQ-5D health dimensions were assigned. Similarly, for each six month period, median values of CD4+ lymphocyte count and log_10_-transformed HIV plasma viral load were assigned for each patient. The number of hospital admissions and number of emergency department visits for each patient were separately summed by half year. The date of death was assigned according to adjudication by the study clinician (WCM) of death dates from two sources: a clinic registry and the Social Security death index. For analyses pertaining to survival, follow up times end on the death date with censoring of remaining patients on the earlier of the date of the last encounter at the study medical center or the end of the study period (30 June 2000). For analyses pertaining to hospital discharges and emergency department utilization, follow up ended on the date of the last encounter at the study medical center or the end of the study period (30 June 2000). For half years in which no encounters at the study medical center were documented, patients were not considered to be at risk for study outcomes by using discontinuous intervals of risk[3]. As a result, patients contribute only those half years to the analysis in which they had at least one clinic visit.

To estimate the percent variance of VAS explained by responses (R^2^) to the five EQ-5D health dimensions, VAS was regressed on the five reference cell coded [4] EQ-5D health dimensions. This analysis was conducted separately for the first five half years of follow up to determine if variance explained decayed with repeated administration of EQ-5D.

To estimate the criterion-related validity of EQ-5D VAS when compared with CD4+ lymphocyte count and HIV plasma viral load, we compared, for the first half year measurement, median VAS scores across categories of CD4+ lymphocyte count (<50, 50–199, ≥ 200 cells/mm^3^) using a Kruskal Wallis test and compared median VAS scores across categories of HIV plasma viral load (< 1,000, ≥ 1,000 copies/mL) using a Wilcoxon rank sum test. We also report receiver operating characteristic (ROC) curve area[5] for EQ-5D VAS as test measure and, as criterion measures, binary coded CD4+ lymphocyte count (<50, ≥50) and plasma viral load (< 1,000, ≥ 1,000 copies/mL). The values of these ROC curve areas represent a measure of how well the VAS can discriminate between low and high values (<50, ≥50) of CD4+ lymphocyte count and between low and high (< 1,000, ≥ 1,000) values of viral load. Values for the areas close to one indicate excellent ability to discriminate, whereas values close to 0.50 indicate close to chance ability to discriminate between the groups.

A predicted VAS (pVAS) was estimated in a random effects linear regression model[6] by regressing the five EQ-5D health states on VAS using indicator coding of the health states. These random effects linear regression models allow for a different intercept for each patient, and the intercepts are assumed to be normally distributed. pVAS may be interpreted as a weighted average of the 5 health dimension scores summarized in a single metric. Survival models which model time to death were fit using Cox modelling incorporating EQ-5D, CD4 count, and HIV plasma viral load as time-varying covariates. It was confirmed that the proportional hazards assumption was met for all covariates included in the Cox models using log(t) by covariates interactions[7]. Hospitalization and ED rate Poisson regression models were estimated using population-averaged generalized estimating equations (GEE)[8,9] with the same time varying covariates. Generalized estimating equation models carry fewer assumptions than other parametric models and focus on marginal effects. Opposite to random effects models where estimated effects of covariates are conditional on subject-specific effects, in a GEE model they relate to effects averaged over individuals. For half years in which no CD4 or viral load was recorded but during which one or more medical encounters occurred at the medical center, the last observation on these laboratory measures was carried forward. The modelling approach for each of the three dependent variables (survival, hospital discharges, and emergency department visits) was to examine CD4 and viral load jointly-adjusted effects of each EQ-5D health dimension, VAS, pVAS, and the health change item. Effects are presented as adjusted hazard ratios (aHR) for time to death and as adjusted incidence rate ratios (aIRR) for ED utilization and hospitalization. It should be noted that although these models examine the adjusted effects of each predictor taken one at a time, the model including pVAS may be interpreted to reflect the joint effect of the five EQ-5D health dimensions summarized as their weighted average. CD4 count was modelled as a time varying covariate using reference cell coding for 3 categories (<50, 50–199, ≥ 200). Similarly, HIV viral load was modelled as a time varying covariate with 2 categories (< 1000, ≥ 1000 copies/ml). Tests of significance are not adjusted for multiple comparisons. Statistical analyses were performed using Stata 9.2 (Stata Corporation, College Station, TX). This research was approved by the University of California San Diego Human Subjects Committee (Project No. 040394).

## Results

Between January 1996 – June 2000, 965 patients met eligibility criteria (Table 1). The study population was predominantly male (88%) with HIV transmission risk factor men having sex with men (59%) and antiretroviral therapy experienced (59%). By race/ethnicity, 34% were either Black or Hispanic. Median absolute CD4 and log_10_-HIV plasma viral load were 233 and 4.6, respectively. Median time at risk was 1.2 years. The median number of half years in which at least one EQ-5D score was documented was 3 (range: 1–9). Forty seven deaths occurred over the 4.5 year study period. Cumulatively over the study period, the percent of patients with 0, 1, and ≥2 emergency department visits was 60%, 24%, and 16%, respectively. The percent of patients with 0, 1, and ≥ 2 admissions was 61%, 23%, and 16%, respectively.

**Table 1:** Baseline Characteristics of Cohort (n = 965)

Characteristic	Distribution
Sex [n (%)]	
Female	117 (12%)
Male	848 (88%)
Age [mean (range)]	37 (18 – 67)
Transmission Risk Factor [n (%)]	
MSM, not IDU	567 (59)
IDU	189 (20)
Heterosexual	160 (17)
Other/Unknown	49 (5)
Race/Ethnicity [n (%)]	
Black	139 (14)
Hispanic	193 (20)
White	559 (58)
Other/Unknown	74 (8)
Prior Antiretroviral Treatment [n (%)]	
Experienced	571 (59)
Naive	394 (41)
Absolute CD4 [mean (std dev)]	233 (337)
log_10 _HIV Plasma Viral Load [mean (std dev)]	4.6 (1.7)

### Variance explained analyses

The variance in VAS scores explained (R^2^) by the five indicator coded EQ-5D health dimensions over the first five half-years of observations was: 39% (n = 915), 47% (n = 735), 48% (n = 511), 56% (n = 348), and 55% (n = 248), respectively. The stability of these estimates with extended follow up suggests that there was no appreciable decay in the meaningfulness of patient responses with repeated administration of the instrument.

### Criterion related validity

**Figure 1:**
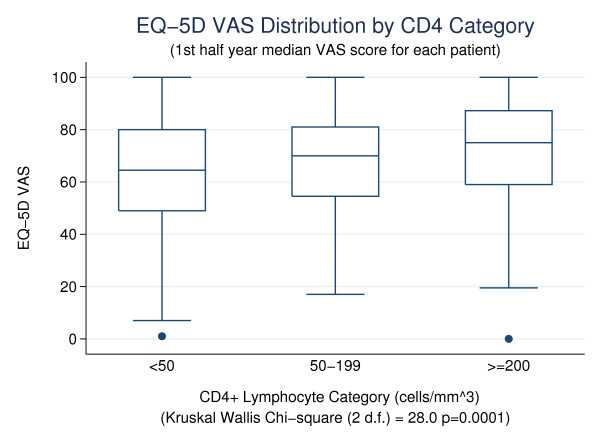
EQ-5D VAS Distribution by CD4 Category (1^st ^half year median VAS score for each patient).

Median EQ-5D VAS scores for the first half year measurement period varied significantly by CD4+ lymphocyte category (see Figure 1) and were borderline significant by HIV plasma viral load. Median EQ-5D VAS scores were 64.5, 70, and 75 for CD4 < 50, 50–199, ≥ 200 cells/mm^3^, respectively (Kruskal Wallis χ^2 ^(2 d.f.) = 28.0, p = 0.001). ROC area for the CD4 criterion (<50, ≥ 50 cells/mm^3^) was 0.614 (95% CI: 0.566 – 0.662). Median VAS scores were 70 and 75.5 for plasma viral load categories ≥ 1000 and < 1000 copies/mL, respectively (Wilcoxon rank sum z = 1.90, p = 0.057. ROC area for the same plasma viral load criteria was 0.555 (95% CI: 0.506 – 0.604).

### Survival outcome

Table 2 presents CD4 and plasma viral load adjusted effects of each EQ-5D health dimension score, VAS, pVAS, and health change on mortality. Each covariate except anxiety/depression was significantly associated with mortality with evidence of a "dose-response" relationship for increasing scores on each of the 4 remaining EQ-5D health dimensions and on the health change item. In addition, higher scores on VAS and on the weighted average of the five EQ-5D health dimensions (pVAS) was significantly (p < 0.05) predictive of survival, with adjusted hazard ratios (aHR) for death of 0.73 and 0.66, respectively. In two separate Cox models of time to death that adjusted simultaneously for CD4+ lymphocyte category, plasma viral load, and either VAS or pVAS, the additional health change item had no independent effect (p = 0.14 and p = 0.14, respectively). Of note, some of the confidence intervals are relatively wide due to a combination of two aspects: 1) Some categories are reported rarely (e.g. unable to wash or dress for self care) and 2) The outcome (death) is relatively rare (47 of all patients with EQ-5D data).

**Table 2:** CD4 and Viral Load Adjusted Hazard Ratios (HR) of EQ-5D and Health Change in Cox Model of Survival

Measure_1_	Half yrs/n/deaths_2_	Adjusted_4_		
		HR	95% CI	p-value

**Mobility**	3006/953/47			<0.001
No problems	1908/./4	1.0		
Some problems	1052/./36	10.0	3.5–28.4	<0.001
Confined to bed	46/./7	27.0	7.7–94.9	<0.001
**Self-Care**	3007/954/47			<0.001
No problems	2421/./13	1.0		
Some problems	547/./26	5.0	2.5–10.0	<0.001
Unable to wash or dress	39/./8	14.1	5.5–36.0	<0.001
**Usual Activities**	3008/954/47			<0.001
No problems	1638/./5	1.0		
Some problems	1249/./27	4.3	1.6–11.3	<0.001
Unable to perform	121/./15	17.3	6.1–49.4	<0.001
**Pain/Discomfort**	3007/954/45			0.001
None	1085/./3	1.0		
Moderate	1609/./26	3.7	1.1–12.4	0.032
Extreme	313/./16	10.9	3.1–37.6	<0.001
**Anxiety/Depression**	3005/954/46			0.086
None	1389/./14	1.0		
Moderate	1365/./25	1.5	0.8–2.9	0.22
Extreme	251/./7	2.8	1.1–7.1	0.028
**VAS_3 _(per 10 units)**	2987/950/44	0.73	0.63–0.83	<0.001
**pVAS (per 10 units)**	2994/948/45	0.66	0.56–0.77	<0.001
**Health change**	3006/955/46			<0.001
Better	761/./5	1.0		
Much the same	1722/./15	1.2	0.4–3.3	0.74
Worse	523/./26	4.6	1.7–12.2	0.002

1. Items 1–5 (Mobility, Self Care, Usual Activities, Pain/Discomfort, Anxiety/Depression) are EQ-5D health states. Health change is not part of EQ-5D. EQ-5D health states, VAS, health change, CD4 category, and HIV-1 plasma viral load are modelled as time-varying covariates.

2. Number of half years/number of patients/number of deaths. Note, the number of patients is not provided for individual categories as half years from the same patient can occur in multiple categories.

3. EQ-5D Visual Analog Scale (VAS) rating of global health status (0–100); pVAS (predicted VAS; see Methods)

4. Each covariate is adjusted only for CD4 category (<50, 50–199, ≥200) and plasma HIV-1 viral load (<1,000, ≥1,000 copies), not for the other listed health status items. CD4 category and plasma HIV-1 viral load are modelled as time-varying covariates.

### Emergency department utilization outcome

Each of the examined covariates was significantly (p < 0.05) associated with emergency department utilization, but the relationships were more complex than those observed for the survival outcome (Table 3). For mobility and self-care, those with "some problems" demonstrated approximately twice the utilization of those with either "no problems" or those who were either confined to bed or unable to wash/dress. Higher scores on the pain item were incrementally predictive of higher visit rates (aIRR 1.7_moderate_, 2.7_extreme_) and those with "extreme" anxiety or depression had higher (aIRR 2.0) emergency utilization than those with no reported anxiety or depression. Higher scores on VAS and pVAS, both reflecting better overall health, were associated with lower emergency department visit rates. Finally, those rating their current health as worse than a year previously had nearly twice the rate of ED utilization than those rating their health as better or as unchanged. In two separate models of ED utilization that adjusted simultaneously for CD4+ lymphocyte category, plasma viral load, and either VAS or pVAS, the additional health change item was independently predictive of the outcome (p = 0.003 and p = 0.02, respectively).

**Table 3:** CD4 and Viral Load Adjusted Incidence Rate Ratios (IRRs) for Emergency Department Visit Rate in Poisson Models

Measure_1_	Adjusted_3_		
	IRR	95% CI	p-value

**1. Mobility**			<0.001
No problems	1.0		
Some problems	2.0	1.7–2.3	<0.001
Confined to bed	1.0	0.6–1.8	0.90
**2. Self-Care**			<0.001
No problems	1.0		
Some problems	1.9	1.6–2.2	<0.001
Unable to wash or dress	1.2	0.7–1.9	0.56
**3. Usual Activities**			<0.001
No problems	1.0		
Some problems	1.8	1.5–2.1	<0.001
Unable to perform	1.9	1.5–2.6	<0.001
**4. Pain/Discomfort**			<0.001
None	1.0		
Moderate	1.7	1.5–2.1	<0.001
Extreme	2.7	2.2–3.4	<0.001
**5. Anxiety/Depression**			<0.001
None	1.0		
Moderate	1.6	1.4–1.9	<0.001
Extreme	2.0	1.6–2.5	<0.001
**VAS_2 _(per 10 units)**	0.86	0.83–0.89	<0.001
**pVAS (per 10 units)**	0.79	0.75–0.82	<0.001
**Health change**			<0.001
Better	1.0		
Much the same	1.1	0.9–1.3	0.30
Worse	1.9	1.5–2.3	<0.001

1. Items 1–5 (Mobility, Self Care, Usual Activities, Pain/Discomfort, Anxiety/Depression) are EQ-5D health states. Health change is not part of EQ-5D. EQ-5D health states, VAS, health change, CD4 category, and HIV-1 plasma viral load are modelled as time-varying covariates.

2. EQ-5D Visual Analog Scale (VAS) rating of global health status (0–100); pVAS (predicted VAS; see Methods)

3. Each covariate is adjusted only for CD4 category (<50, 50–199, ≥200) and plasma HIV-1 viral load (<1,000, ≥1,000 copies), not for the other listed health status items. CD4 category and plasma HIV-1 viral load are modelled as time-varying covariates.

### Hospital discharge rate outcome

For the hospitalization rate outcome (Table 4), each of the examined covariates was predictive. Those with the highest scores on mobility, self-care, usual activities, and pain/discomfort had 2.3 to 3.5 times the rates of hospitalization than those with no impairment in those health dimensions. Those with extreme anxiety or depression had a 50% higher rate than those with no anxiety or depression. Both global health indicators (VAS and pVAS) showed significant relationships in the predicted direction with hospitalization with adjusted aIRRs of 0.85 and 0.79, respectively. Those who rated their current health as worse than a year previously had twice the hospitalization rate of those who rated it as better. In two separate models of hospitalization rate that adjusted simultaneously for CD4+ lymphocyte category, plasma viral load, and either VAS or pVAS, the additional health change item was independently predictive of the outcome when VAS was included (p = 0.02), but not when pVAS was included (p = 0.09).

**Table 4:** CD4 and Viral Load Adjusted Incidence Rate Ratios (IRRs) for Hospital Discharge Rate in Poisson Models

Measure_1_	Adjusted_3_		
	IRR	95% CI	p-value

**Mobility**			<0.001
No problems	1.0		
Some problems	2.3	2.0–2.8	<0.001
Confined to bed	2.9	2.1–4.0	<0.001
**Self-Care**			<0.001
No problems	1.0		
Some problems	2.1	1.8–2.4	<0.001
Unable to wash or dress	2.3	1.7–3.2	<0.001
**Usual Activities**			<0.001
No problems	1.0		
Some problems	2.2	1.9–2.7	<0.001
Unable to perform	3.5	2.7–4.5	<0.001
**Pain/Discomfort**			<0.001
None	1.0		
Moderate	1.9	1.6–2.3	<0.001
Extreme	3.1	2.4–3.9	<0.001
**Anxiety/Depression**			0.01
None	1.0		
Moderate	1.1	0.9–1.3	0.31
Extreme	1.4	1.1–1.8	0.002
**VAS_2 _(per 10 units)**	0.85	0.82–0.88	<0.001
**pVAS (per 10 units)**	0.79	0.75–0.82	<0.001
**Health change**			<0.001
Better	1.0		
Much the same	1.2	1.0–1.5	0.032
Worse	2.0	1.6–2.5	<0.001

1. Items 1–5 (Mobility, Self Care, Usual Activities, Pain/Discomfort, Anxiety/Depression) are EQ-5D health states. Health change is not part of EQ-5D. EQ-5D health states, VAS, health change, CD4 category, and HIV-1 plasma viral load are modelled as time-varying covariates.

2. EQ-5D Visual Analog Scale (VAS) rating of global health status (0–100); pVAS (predicted VAS; see Methods)

3. Each covariate is adjusted only for CD4 category (<50, 50–199, ≥200) and plasma HIV-1 viral load (<1,000, ≥1,000 copies), not for the other listed health status items. CD4 category and plasma HIV-1 viral load are modelled as time-varying covariates.

## Discussion

This study has demonstrated that a brief, non-disease specific measure of health related quality of life, when administered routinely to adults under care for HIV infection, captures prognostic information independent of current CD4+ lymphocyte count and HIV plasma viral load. In addition, we confirmed previous observations[10] that the EQ-5D VAS score has the potential to discriminate among patients varying in CD4+ lymphocyte count. The EQ-5D also has high acceptability in a busy HIV clinic, even when administered at every routine clinic visit by medical assistants as part of vital sign documentation. Meaningfulness of patient responses on repeat administration seems not to decay when assessed by variance in VAS scores explained by responses on the EQ-5D health dimensions.

HR-QOL is a multidimensional construct, the components of which have been conceptualized as encompassing physiological factors, symptom status, functional health, general health perceptions, and overall quality of life[11] and this conceptual model has received some empirical validation recently in a retrospective analysis of an HIV health care cohort[12]. There have been a number of reviews since the early 1990s of HR-QOL measures administered to patients with HIV infection, and no consensus has emerged on a clearly preferred instrument for use either in clinical trials or in clinical care [13-20]. However, a number of both disease-specific and generic measures have been advocated for use in specific populations and settings. The most well validated ones have been reviewed most recently by Clayson et al[13] who among generic measures considered the EQ-5D, Health Utilities Index (HUI), and SF-36, and among HIV-specific measures, reviewed the following six measures: Medical Outcomes Study HIV Health Survey (MOS-HIV), Functional Assessment of HIV Infection (FAHI), Multidimensional QOL for Persons Living with HIV/AIDS (MQoL-HIV), HIV/AIDS-Targeted Quality of Life Instrument (HAT-QoL), Living With HIV Scale (LWH), and General Health Self-Assessment (GHSA). These nine instruments were reviewed for possible use in clinical trials on the basis of four review criteria: (1) content validity for physical function, social/role function and mental health/emotional well-being, (2) practicality (self administered taking ≤ 15 minutes with ≤ 50 items), (3) psychometric properties (dimensionality, reliability, validity, and responsiveness), and (4) the availability of normative data and/or population-based preference weights. Clayson and co-workers concluded that, although there is no one best HR-QOL measure for use in HIV/AIDS clinical trials, three generic (EQ-5D, SF-36, HUI) and two HIV-targeted candidate measures (FAHI, MOS-HIV) appear to be more favorable than others for consideration.

Responses to the five EQ-5D health dimensions define 243 possible health states for which general population preference weights have been derived for U.S., European, Japanese, and African samples. The population-specific preference weights for EQ-5D health states can be transformed into summary index scores (EQ-5D_index_) suitable for use in cost-utility studies. In a cross sectional study of a sample of HIV-infected patients, Delate and Coons demonstrated that EQ-5D health index scores and VAS discriminated between patients with CD4 ≤ 200 cells/mm^3 ^and CD4>200 cells/mm^3 ^and between those with HIV plasma viral load ≤ 30,000 copies/mL and > 30,000 copies/mL[10]. In an AIDS clinical trial setting with all patients entering with CD4<100 cells/mm^3^, Wu et al. demonstrated that both the EQ-5D Index and EuroQol VAS correlated with MOS-HIV mental health and physical health summary scores[21]. In the same study, the investigators evaluated the responsiveness of EQ-5D to the development of adverse events (AEs) and opportunistic infections (OI) during the trial. The EQ-5D was less responsive than MOS-HIV pain and physical health scores to AEs. However, the EuroQol VAS was more responsive to OI induced change than the MOS-HIV physical health score. In a previous study of the UCSD Owen Clinic cohort, we observed, in a sample with median CD4 = 117 cells/mm^3^, that scores on five EQ-5D health dimensions explained 46% of the variance in the patients' concomitant VAS ratings. Of the possible 243 possible EQ-5D health states, 83 were observed in the patient sample of 530[22].

A global single item rating of health change such as was included in this study is one type of an anchor-based method for determining health change. Such measures have the advantages that they are easy to ascertain and take into account a variety of information from the patient's perspective. However, like all single item measures, they may have limited precision and reliability and may yield varying results when current health is compared to other anchor states[23]. Studies have shown that responses to global health transition items may be disproportionately influenced by the current health state and may not adequately incorporate their state at the reference time point[24,25]. Nonetheless, health transition items may complement and at times be more sensitive to change than multi-item measures of current health state[26]. The addition of a single item health change measure in our study was shown to contribute independent prognostic information when compared to the EuroQol VAS and pVAS. Future analyses of the dataset will explore whether changes in sequential EQ-5D responses are correlated with patients' retrospective global assessment of health change. These future analyses will also contribute to an examination of whether the EQ-5D is subject to a "ceiling effect" in this population, a phenomenon that limits the ability to detect change when baseline scores are high and which has been reported when EQ-5D has been used in general population studies[27].

Inference from this research is subject to a number of limitations. First, although assessment of the mortality outcome is robust, there may have been some hospitalizations and ED visits outside our health care system that were not captured in our data sources. We believe that this outside utilization to be minor because the study medical center is the primary referral center for HIV care in the civilian and public sectors in San Diego. Second, although it is clinic policy to administer the EQ-5D at all primary care and urgent visits, when patients come in severely ill, the survey may not be administered. This bias would tend to attenuate an association between EQ-5D scores and morbid outcomes, but our analysis nonetheless showed easily detectable effects for all three outcome measures. Third, previous evaluations of EQ-5D have used raw scores as the unit of analysis. Our study averaged scores over calendar half years in order to provide more stable estimates of health status within half year periods. We acknowledge that this approach may limit the comparability of the findings to studies using individual administrations of EQ-5D as the unit of analysis. Fourth, because of the averaging of scores within half years, there is some temporal ambiguity in interpreting possible causal associations between EQ-5D scores and the rates of ED use and hospitalization. That is, within a given half year, some of the EQ-5D scores were ascertained after an outcome event had taken place and could not therefore be considered predictive of an antecedent outcome. On the other hand, to the extent that events occurred in future half years, a predictive association would be validly demonstrated. This is not a limitation for the mortality outcome.

## Conclusion

Despite these limitations, we conclude that both the EQ-5D and health change item contribute meaningful prognostic information for three important health care outcomes for adults under care for HIV infection. The prognostic value of these measures will be of use in epidemiological studies but not necessarily evident in prediction of individual patient outcomes. The research has confirmed previous support for the criterion-related validity of EQ-5D when assessed in relation to CD4+ lymphocyte category and plasma viral load. Finally, we have some confidence that repeated administration of the instrument in a clinical care setting does not result in decay of the meaningfulness of responses when assessed using a criterion of percent variance explained.

## Competing interests

The author(s) declare that they have no competing interests.

## Authors' contributions

WCM designed the study, performed initial statistical analyses, and drafted the manuscript. SM participated in the analysis plan, performed final statistical analyses, and contributed substantively to the submitted manuscript preparation. Both authors read and approved the final manuscript.
